# Regulation of Chemokine CCL5 Synthesis in Human Peritoneal Fibroblasts: A Key Role of IFN-**γ**

**DOI:** 10.1155/2014/590654

**Published:** 2014-01-09

**Authors:** Edyta Kawka, Janusz Witowski, Nina Fouqet, Hironori Tayama, Thorsten O. Bender, Rusan Catar, Duska Dragun, Achim Jörres

**Affiliations:** ^1^Department of Nephrology and Medical Intensive Care, Charité-Universitätsmedizin Berlin, Campus Virchow-Klinikum, Augustenburger Platz 1, 13353 Berlin, Germany; ^2^Department of Pathophysiology, Poznan University of Medical Sciences, Swiecickiego 6, 60-781 Poznań, Poland

## Abstract

Peritonitis is characterized by a coordinated influx of various leukocyte subpopulations. The pattern of leukocyte recruitment is controlled by chemokines secreted primarily by peritoneal mesothelial cells and macrophages. We have previously demonstrated that some chemokines may be also produced by human peritoneal fibroblasts (HPFB). Aim of our study was to assess the potential of HPFB in culture to release CCL5, a potent chemoattractant for mononuclear leukocytes. Quiescent HPFB released constitutively no or trace amounts of CCL5. Stimulation of HPFB with IL-1**β** and TNF-**α** resulted in a time- (up to 96 h) and dose-dependent increase in CCL5 expression and release. IFN-**γ** alone did not induce CCL5 secretion over a wide range of concentrations (0.01–100 U/mL). However, it synergistically amplified the effects of TNF-**α** and IL-1**β** through upregulation of CCL5 mRNA. Moreover, pretreatment of cells with IFN-**γ** upregulated CD40 receptor, which enabled HPFB to respond to a recombinant ligand of CD40 (CD40L). Exposure of IFN-**γ**-treated HPFB, but not of control cells, to CD40L resulted in a dose-dependent induction of CCL5. These data demonstrate that HPFB synthesise CCL5 in response to inflammatory mediators present in the inflamed peritoneal cavity. HPFB-derived CCL5 may thus contribute to the intraperitoneal recruitment of mononuclear leukocytes during peritonitis.

## 1. Introduction

Peritoneal dialysis (PD) is an effective alternative to haemodialysis as a life-saving renal replacement therapy for patients with chronic kidney disease. However, the technique may fail as a result of repeated episodes of peritoneal infection that lead to peritoneal membrane damage and loss of its ultrafiltration capacity [1, 2]. The peritoneal cavity contains normally variable numbers of resident leukocytes, predominantly macrophages but also lymphocytes (mostly memory T cells), dendritic, and natural killer (NK) cells [3]. In contrast, acute peritonitis is characterized by a massive influx of polymorphonuclear leukocytes (PMN) [4]. PMN ingest invading microorganisms and then are gradually cleared and replaced by mononuclear cells (monocytes, macrophages, and lymphocytes) so that the intraperitoneal homeostasis is restored. The whole process is governed by a complex network of cytokines, growth factors, adhesion molecules, and molecules derived from pathogens and damaged cells [5]. In this respect, chemokines of various classes create chemotactic gradients that mediate migration of specific leukocyte subpopulations into the peritoneal cavity. In early stages of peritonitis proinflammatory cytokines (TNF-*α* and IL-1*β*) derived from resident macrophages induce the expression of CXC chemokines that attract PMN. Then, upon the influence of IFN-*γ* and IL-6, the pattern of chemokine expression changes so that CC chemokines predominate and mediate mononuclear cell recruitment [6].

During peritonitis chemokines are produced mainly by cytokine-stimulated mesothelial cells that cover the peritoneal membrane. However, in recent years it has become clear that fibroblasts embedded in peritoneal interstitium act not only as structural cells but may also serve as an important source of chemokines [7]. Thus, by producing various chemokines fibroblasts may modify both the intensity and the duration of the inflammatory response [8]. We have previously demonstrated that human peritoneal fibroblasts (HPFB) generate significant quantities of CXC chemokines that attract and promote survival of PMN during PD-associated peritonitis [9]. Moreover, HPFB are able to produce CCL2, which belongs to CC chemokines and acts mainly as a monocyte chemoattractant [7].

CCL5 (CC-chemokine ligand 5) is another member of the CC chemokine family. First identified in T cells and designated RANTES (Regulated upon Activation, Normal T cell Expressed and Secreted), CCL5 is an 8 kDa protein consisting of 68 amino acids [10]. In addition to lymphocytes, it was found to be also produced by stromal cells. Acting through three types of chemokine receptors (CCR1, CCR3, and CCR5), CCL5 is broadly chemoattractive for T lymphocytes and NK cells, monocytes, basophils, and eosinophils [11]. Interestingly, once T lymphocytes reach the site of injury and become activated with specific antigens, they start producing large amounts of CCL5 after 3–5 days, which maintains and amplifies the immune response. Although there is a great deal of overlapping in biological activities of CC chemokines, the experimental studies in mice demonstrate that CCL5 deficiency is associated with impaired T-cell proliferation and function [12]. This observation indicates that CCL5 is uniquely essential for T-cell recruitment in vivo. Therefore, in the present study we have analysed how proinflammatory cytokines known to be present in the inflamed peritoneum regulate CCL5 production by peritoneal fibroblasts.

## 2. Materials and Methods

Unless stated otherwise, all chemicals were from Sigma-Aldrich (St Louis, MO, USA) and all culture plastics were Falcon from Becton Dickinson (Heidelberg, Germany). Cell culture media and foetal calf serum (FCS) were from Invitrogen/Life Technologies (Darmstadt, Germany), and other cell culture reagents were from Biochrom AG (Berlin, Germany). Human recombinant cytokines and anticytokine antibodies were from R&D Systems (Wiesbaden, Germany). IFN-*γ* specific activity was 2 × 10^4^ WHO standard units per 1 *μ*g protein (1 U/mL = 50 pg/mL).

### 2.1. Isolation and Culture of Human Peritoneal Fibroblasts (HPFB)

HPFB were isolated from the specimens of apparently normal omentum obtained from consenting patients undergoing elective abdominal surgery. The tissue was treated with four rounds of digestion with trypsin, as described in detail elsewhere [13]. HPFB were identified by spindle-shape appearance, formation of parallel arrays and whorls at confluence [13], and positive immunostaining for fibroblast specific protein 1 (FSP-1) [14]. Cells were propagated in Ham's F12 culture medium supplemented with penicillin (100 U/mL), streptomycin (100 *μ*g/mL), hydrocortisone (0.4 *μ*g/mL), and 10% (v/v) FCS. HPFB cultures were maintained at 37°C in a humidified atmosphere of 95% air and 5% CO_2_. All experiments were performed using cells from the first 3 passages and with cells derived from separate donors. Before the experiments, cells were rendered quiescent by reducing FCS concentration to 0.1% for 48 hours. Cells were then treated as specified in the figure legends. After the exposure, the supernatants were collected and stored in aliquots at −80°C until assayed.

### 2.2. CCL5 Protein Measurement

Concentrations of CCL5 protein secreted by HPFB were measured with the DuoSet Immunoassay Development Kit (R&D Systems). The assay was designed and performed according to the manufacturer's instructions. Sensitivity of the assay was 5 pg/mL.

### 2.3. Gene Expression Analysis

Expression of CCL5 gene was assessed with reverse transcription (RT) and PCR. Total RNA was extracted with RNA Bee (Tel-Test, Friendswood, TX, USA), purified with the RNeasy kit (Qiagen, Hamburg, Germany), and reverse transcribed into cDNA with random hexamer primers, as described in [15]. Conventional semiquantitive PCR was carried out essentially as described by Robson et al. for CCL5 and *β*-actin [16], and by Abdel-Haq et al. for CD40 [17]. Precise quantitation of CCL5 mRNA was performed by real-time PCR. The reactions were carried out in Roche LightCycler II using 20 ng of cDNA and FastStart DNA Master SYBR Green I reagents according to the manufacturer's instructions (Roche Applied Science, Indianapolis, IN, USA). Primer pairs (TIB Molbiol, Berlin, Germany) spanned an intron to eliminate potential amplification of contaminating genomic DNA. The following primers were used: CCL5 (GenBank NM_002985.2) forward, GAGTATTTCTACACCAGTGGCAAG; reverse, TCCCGAACCCATTTCTTCTCT; GAPDH (GenBank NM_002046.4) forward, TGATGACATCAAGAAGGTGGTGAAG; reverse, TCCTTGGAGGCCATGTGGGCCAT. Cycle parameters were as follows: denaturation at 95°C for 10 s, annealing at 63°C for 5 s, and elongation at 72°C for 20 s for 40 cycles. Melting curve analyses were performed from 60°C to 95°C in 0.5°C increments. Quantitative PCR data for CCL5 were normalized based on GAPDH transcript levels. Run data were analysed by “second derivative maximum” with the quantification program Quant versions 2.7 and 3.0.

### 2.4. Statistical Analysis

Data are presented as mean ± SEM of the results obtained in independent experiments with cells from different donors. Statistical analyses were carried out using GraphPad Prism 5.00 software (GraphPad Software Inc., La Jolla, CA, USA). The data were compared with repeated measures analysis of variance with Newman-Keuls modification or the paired *t*-test, as appropriate. A *P* value of <0.05 was considered significant. Significant differences compared with appropriate controls were denoted with asterisks: *P < 0.05; **P < 0.01; ***P < 0.001.

## 3. Results

### 3.1. Induction of CCL5 Production in HPFB by IL-1*β* and TNF-*α*

**Figure 1:**
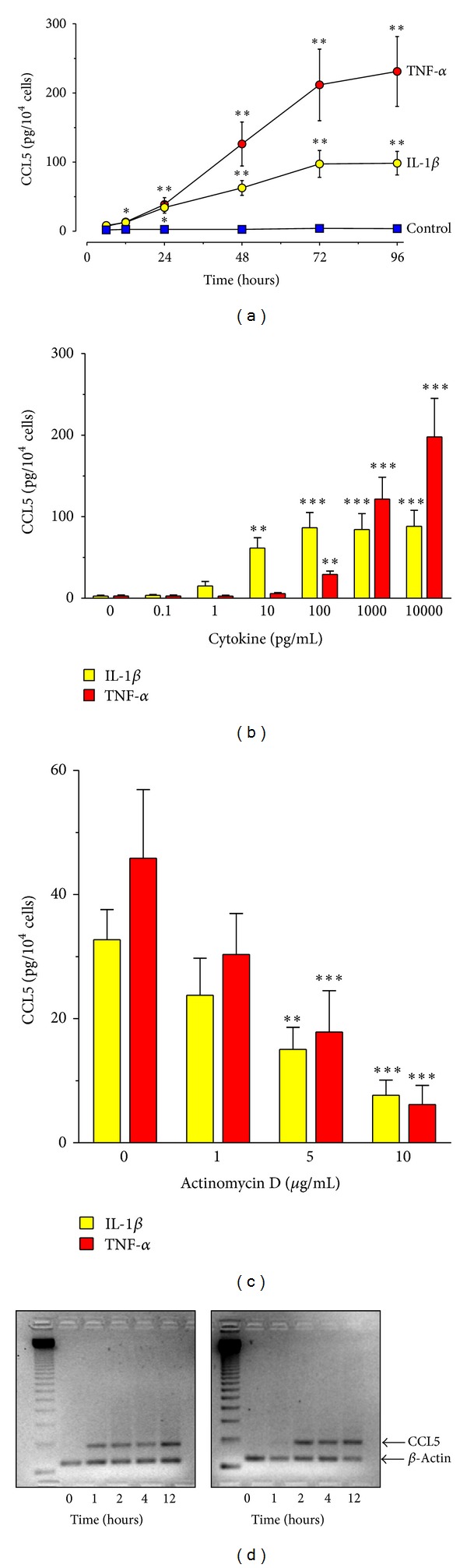
Effect of recombinant IL-1*β* and TNF-*α* on CCL5 expression and release by HPFB. Cells were exposed to either IL-1*β* or TNF-*α*. The data were derived from experiments with cells isolated from separate donors. (a) Kinetics of IL-1*β*-induced (1000 pg/mL) or TNF-*α*-induced (1000 pg/mL) CCL5 secretion (*n* = 6); (b) dose effect of IL-1*β* or TNF-*α*. Cells were stimulated for 48 hours (*n* = 6); (c) effect of actinomycin D on CCL5 release by HPFB. Cells were pretreated for 1 hour with actinomycin D and then exposed to either IL-1*β* or TNF-*α* (both at 1000 pg/mL) for 24 hours (*n* = 6); (d) time effect of IL-1*β* and TNF-*α* on CCL5 mRNA expression. HPFB were treated with cytokines at 1000 pg/mL for the times indicated. CCL5 mRNA expression was analysed by semiquantitative RT-PCR. Results of a representative experiment of three performed.

The amount of CCL5 released constitutively by quiescent HPFB was barely detectable (Figure 1). In contrast, stimulation of HPFB with recombinant proinflammatory cytokines IL-1*β* and TNF-*α* resulted in a time- and dose-dependent CCL5 secretion. The release of CCL5 in response to IL-1*β* was significantly above the background levels within 12–24 hours of incubation and reached plateau after 72 hours. The time course of CCL5 generation in response to the same concentration of TNF-*α* followed a similar pattern; however, even greater amounts of CCL5 were produced (Figure 1(a)). Experiments assessing the dose effect of cytokines revealed that IL-1*β* was effective already at concentrations as low as 1 pg/mL and the effect reached saturation at 100 pg/mL (Figure 1(b)). TNF-*α* was able to stimulate CCL5 release at concentrations ranging from 100 to 10000 pg/mL.

Pretreatment of HPFB with actinomycin D resulted in a dose-dependent inhibition of cytokine-induced CCL5 secretion, indicating that the stimulatory effects of IL-1*β* and TNF-*α* occurred at the transcriptional level (Figure 1(c)). Indeed, treatment of HPFB with either IL-1*β* or TNF-*α* resulted in a time-dependent upregulation of the CCL5 mRNA signal, as visualized by conventional semiquantitative PCR (Figure 1(d)).

### 3.2. Effect of IFN-*γ* on CCL5 Production by HPFB

**Figure 2:**
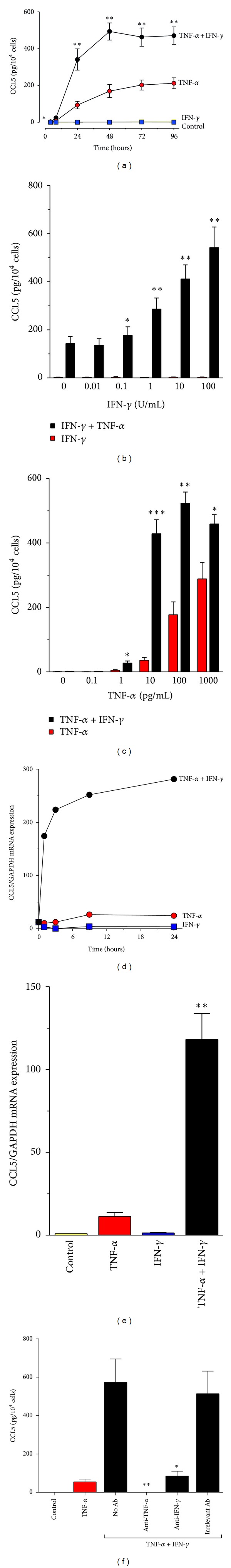
CCL5 induction in HPFB stimulated with TNF-*α* and IFN-*γ*. (a) Kinetics of CCL5 secretion by HPFB treated with TNF-*α* (1000 pg/mL) and IFN-*γ* (25 U/mL) alone or in combination. Asterisks represent a significant difference compared with the predictive additive values at each time point (*n* = 6); (b) dose effect of IFN-*γ* alone or with TNF-*α* (1000 pg/mL; *n* = 7); (c) dose effect of TNF-*α* alone or with IFN-*γ* (25 U/mL; *n* = 5). B and C cells were stimulated for 48 hours. Asterisks represent statistically significant differences compared to the predictive additive values; (d) kinetics of TNF-*α* and IFN-*γ*-induced CCL5 mRNA. Cells were treated with TNF-*α* and/or IFN-*γ* for the times indicated. Results of an exemplary experiment of two performed; (e) magnitude of CCL5 mRNA expression in HPFB treated for 24 hours with TNF-*α* and/or IFN-*γ*. Results of 4 experiments with cells from separate donors. Asterisks represent a significant difference compared to the predictive additive value. D and E cells were treated with TNF-*α* at 1000 pg/mL and IFN-*γ* at 25 U/mL. CCL5 mRNA expression relative to that of GAPDH was quantified with real-time PCR; (f) effect of neutralizing anti-TNF-*α* or anti-IFN-*γ* antibodies on synergistic CCL5 release by HPFB. Cells were incubated with antibodies (all at 1 *μ*g/mL) for 48 h. Asterisks represent a significant difference compared with cells treated with a combination of TNF-*α* (1000 pg/mL) and IFN-*γ* (25 U/mL) in the absence of antibodies (*n* = 4).

IFN-*γ* at concentrations ranging from 0.01 to 100 U/mL did not induce CCL5 production by HPFB. However, it amplified synergistically CCL5 release induced by TNF-*α* (Figure 2) and—to lesser extent—by IL-1*β* (not shown). The effect was time-dependent (Figure 2(a)) and related to the dose of both IFN-*γ* and TNF-*α* (Figures 2(b) and 2(c)). Concentration of IFN-*γ* as low as 0.1 U/mL was capable of amplifying the effect of 1000 pg/mL TNF-*α*. On the other hand, 25 U/mL IFN-*γ* magnified the effect exerted by 10 pg/mL TNF-*α* more than 10-fold.

Although IFN-*γ* alone did not induce CCL5 mRNA, it produced a rapid (within 1 hour) synergistic increase in TNF-*α*-driven CCL5 expression, which persisted over 24 hours (Figure 2(d)). Quantitative assessment showed that CCL5 mRNA expression in response to a combination of TNF-*α* + IFN-*γ* was approximately 10-fold greater than that induced by TNF-*α* alone (Figure 2(e)).

### 3.3. Specificity and Timing of the Effects of IFN-*γ* and TNF-*α* on CCL5 Release by HPFB

Specificity of the combined stimulation by IFN-*γ* and TNF-*α* was verified in experiments using blocking antibodies. Neutralization of IFN-*γ* decreased CCL5 production to a level achieved by treatment with TNF-*α* alone (Figure 2(f)). In turn, anti-TNF-*α* antibodies totally abolished CCL5 secretion in response to TNF-*α* + IFN-*γ*. Control antibody of the same class did not affect CCL5 release. To determine whether the synergistic effect of TNF-*α* and IFN-*γ* was related to the sequence of stimuli, HPFB were incubated in the presence or absence of TNF-*α* or IFN-*γ* for 24 hours, then washed, and stimulated again for further 24 hours (Table 1). These experiments showed some degree of priming with either TNF-*α* or IFN-*γ*. However, the greatest synergy was observed when both cytokines were applied together. Interestingly, the effect of combined stimulation with TNF-*α* and IFN-*γ* for the first 24 hours still persisted during the next 24 hours, even in the absence of cytokines.

### 3.4. Effect of CD40 Ligand (CD40L) on CCL5 Induction in HPFB

**Figure 3:**
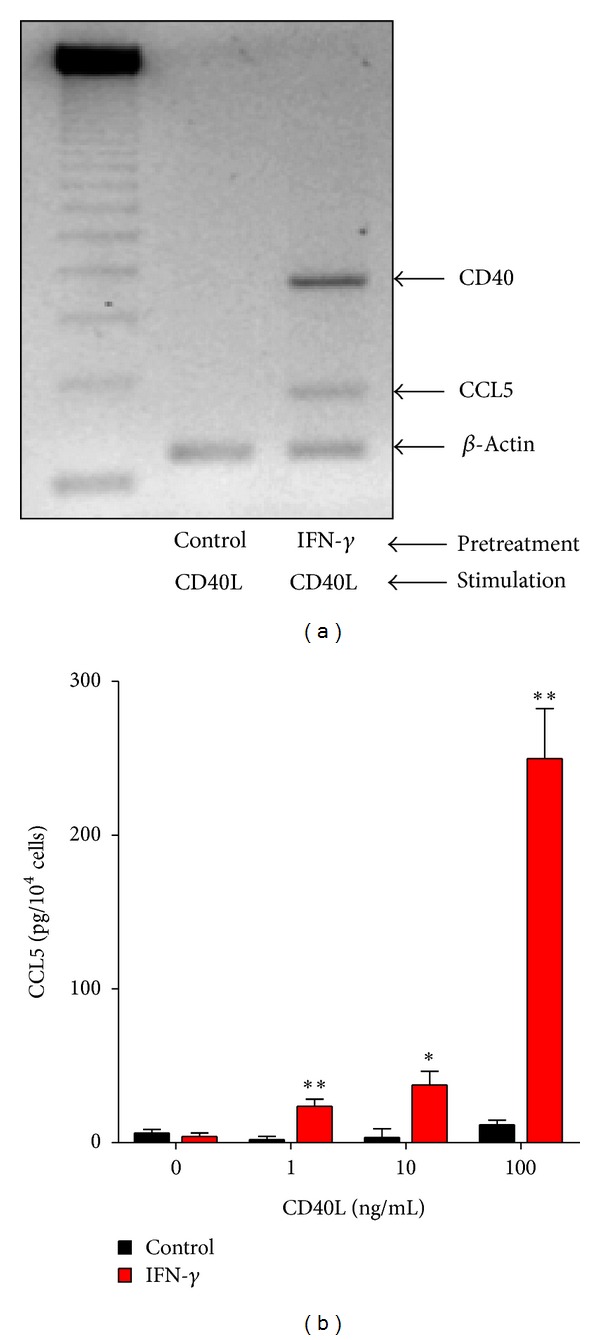
Effect of preexposure to IFN-*γ* on CD40L-induced CCL5 expression and release by HPFB. Cells were pretreated for 48 hours either with control medium or IFN-*γ* (100 U/mL). After that cells were stimulated with CD40L for the next 24 hours. (a) Expression of mRNA for CD40 and CCL5 was assessed by conventional RT-PCR. Results of a representative experiment of two performed. (b) CCL5 release was measured in HPFB cultures established from 5 separate donors. Asterisks represent a significant difference compared to cells not exposed to IFN-*γ*.

CD40L, a member of the TNF-*α* family, is expressed by mononuclear cells infiltrating the peritoneum during peritonitis [18]. We have therefore examined if CD40L is able to induce CCL5 in HPFB. It turned out that CD40L had almost no effect in control cells but stimulated dose-dependent CCL5 release in HPFB pretreated with IFN-*γ* (Figure 3). We have then used PCR to assess the expression in HPFB of CD40, a receptor for CD40L. Unstimulated cells did not express CD40 mRNA; however its presence could be detected following the treatment with IFN-*γ*. Accordingly, subsequent stimulation with CD40L induced CCL5 mRNA expression in cells pretreated with IFN-*γ*.

## 4. Discussion

The ability of chemokines to recruit specific leukocyte subpopulations is crucial for controlling the course of inflammatory response. Thus, the regulation of chemokine production is equally important. We have shown that peritoneal fibroblasts produce significant quantities of chemokine CCL5. This observation is in keeping with the view of fibroblasts as sentinel cells providing address codes for migrating leukocytes [19]. It has previously been shown that peritoneal mesothelial cells synthesize CCL5 in response to inflammatory cytokines [16, 20, 21]. However, peritonitis may result in serious mesothelial cell damage and exfoliation [22, 23]. The function of peritoneal fibroblasts may then become essential, providing an alternative and/or additional source of chemokines. Although CCL5 production has been demonstrated in fibroblast from other locations, such as pancreas [24], skin [25, 26], gingiva [27], nasal mucosa [28, 29], and synovium [30], it is important to study the function of fibroblasts derived precisely from the tissue of interest. It is because fibroblasts display tissue-specific phenotypes that include different patterns of chemokine expression [31, 32], which may contribute to characteristic composition of leukocyte infiltrates.

Here, we show that HPFB in culture do not release CCL5 constitutively but are capable of producing this chemokine de novo in response to stimulation with proinflammatory cytokines IL-1*β* and TNF-*α*. Of those, TNF-*α* appears to be a more potent stimulus, which is in contrast to its effect on CXC chemokines, whose production in HPFB was found to be induced primarily by IL-1*β* [9]. This differential responsiveness to IL-1*β* and TNF-*α* may provide yet another level of regulation to chemokine release by HPFB.

CCL5 mediates the influx of mononuclear cells, including T cells, which are the main source of IFN–*γ* in the dialysed peritoneum [33]. IFN-*γ* can further amplify CCL5 production through synergistic induction of CCL5 mRNA. Interestingly, IFN-*γ* exerted this effect despite the fact that when acting on its own, it did not stimulate CCL5. Similar results were observed in mesothelial cells [16], synovial fibroblasts [34], endothelial cells [35], and alveolar epithelial cells [36]. In contrast, in mouse macrophages IFN-*γ* was found to directly induce CCL5 [37]. Early induction of CCL5 gene in response to TNF-*α* and IFN-*γ* suggests that the effect is mediated by rapidly activated transcription factors that bind to CCL5 promoter. In this respect, nuclear factor *κ*B (NF-*κ*B) was found to be a chief mediator involved [36, 38]. It may further cooperate with interferon regulatory factors (IRF) [39, 40] and signal transducers and activators of transcription (STATs) [38].

In addition to T-cells, CCL5 attracts also eosinophils. This feature is interesting, as peritoneal eosinophilia may occur in the course of peritoneal dialysis [41] and may be related to exposure of the peritoneal membrane to foreign environment [42]. Interestingly, it has been demonstrated in an animal model of peritoneal dialysis that peritoneal eosinophilia and CCL5 elevation was particularly pronounced after exposure to dialysis fluids regarded as less biocompatible [43].

CCL5-induced leukocyte infiltrate contains T-lymphocytes that express a membrane-bound CD40L [18]. It has been demonstrated that fibroblasts from various sources express no or very little CD40 mRNA; however, it can be upregulated through IFN-*γ* [44]. This effect corresponds to an increase in CD40 cell surface expression [44–46]. We have found that exposure to IFN-*γ* increased CD40 expression in HPFB and made them responsive to CD40L. Ligation of thus induced CD40 by CD40L resulted in increased CCL5 production. Such an effect was observed previously in fibroblasts from inflamed colonic mucosa [47], but also in peritoneal mesothelial cells [45]. The underlying mechanism most likely involves NF-*κ*B, which was shown to be activated by CD40 ligation [46]. CD40L-induced CCL5 may create positive feedback loop that further supports lymphocyte influx. In this respect, it has been shown that increased CD40L expression on peritoneal lymphocytes and macrophages supports the transition to mononuclear cell predominance in the late phase of peritonitis and timely resolution of inflammation [18].

In conclusion, our study demonstrates the great potential of peritoneal fibroblasts to generate CCL5 in response to activation by proinflammatory mediators encountered during peritonitis. By establishing a CCL5 gradient, HPFB may facilitate mononuclear leukocyte recruitment and successful resolution of inflammation. On the other hand, repeated and/or severe episodes of infection may injure the protective mesothelium and expose underlying HPFB to excessive stimulation. In those circumstances, HPFB-derived CCL5 may promote leukocyte infiltration into the peritoneal interstitium, which may lead to prolonged inflammation. In both scenarios HPFB would be actively involved in the cytokine network controlling the course of inflammation.

## Figures and Tables

**Table 1:** Effect of sequential addition of TNF-*α* and IFN-*γ* on CCL5 release by HPFB.

Stimulus 1	Stimulus 2	CCL5 (pg/10^4^ cells)
Medium	Medium	Undetectable
Medium	IFN-*γ*	Undetectable
Medium	TNF-*α*	4 ± 2
Medium	TNF-*α* + IFN-*γ*	24 ± 13
IFN-*γ*	Medium	1 ± 1
IFN-*γ*	TNF-*α*	9 ± 4
TNF-*α*	Medium	6 ± 4
TNF-*α*	IFN-*γ*	9 ± 7
TNF-*α* + IFN-*γ*	Medium	146 ± 20

Cells were incubated with TNF-*α* (1000 pg/mL) and/or IFN-*γ* (25 U/mL) for 24 hours (stimulus 1), washed, and incubated again for the next 24 hours in the presence or absence of these cytokines (stimulus 2). Data were derived from two independent experiments.
